# Strengths, Weaknesses, Opportunities and Threats (SWOT) Analysis of the Implementation of Public Health Policies on HTLV-1 in Brazil

**DOI:** 10.3389/fmed.2022.859115

**Published:** 2022-04-07

**Authors:** Angelica Espinosa Miranda, Carolina Rosadas, Tatiane Assone, Gerson Fernando Mendes Pereira, Antonio Carlos Rosário Vallinoto, Ricardo Ishak

**Affiliations:** ^1^Departamento de Condições Crônicas e Infecções Sexualmente Transmissíveis, Secretaria de Vigilância em Saúde, Ministério da Saúde, Brasília, Brazil; ^2^Departamento de Medicina Social, Universidade Federal do Espírito Santo, Vitória, Brazil; ^3^Section of Virology, Department of Infectious Disease, Faculty of Medicine, Imperial College London, London, United Kingdom; ^4^Laboratório de Investigação Médica em Neurologia, Faculdade de Medicina, Instituto de Medicina Tropical de São Paulo, Universidade São Paulo, São Paulo, Brazil; ^5^Laboratório de Virologia, Instituto de Ciências Biológicas, Universidade Federal do Pará, Belém, Brazil

**Keywords:** HTLV-1, SWOT analysis, public health policies, prevention, control, public policies, transmission

## Abstract

Human T lymphotropic virus 1 (HTLV-1) is a public health issue for most countries and imposes important consequences on patients' health and socioeconomic status. Brazil is one of the global leaders of the public health response to these viruses. The country has challenges to overcome to implement meaningful policies aiming to eliminate HTLV-1/2. An analysis of strengths, weaknesses, opportunities, and threats (SWOT) for the implementation of public health policies on HTLV-1/2 was performed. The strengths identified were the Brazilian Unified Health System (SUS); Brazilian expertise in public health programs successfully implemented; currently available policies targeting HTLV; and strong collaboration with researchers and patient's representative. Lack of awareness about HTLV, insufficient epidemiological data, lack of reference centers for patient care, insufficient availability of confirmatory tests, lack of universal antenatal screening, and absence of cost-effectiveness studies were identified as weaknesses. Some interesting opportunities included the increased interest from international organizations on HTLV, possibility of integrating HTLV into other programs, external funding for research, available online platforms, opportunity to acquire data from HTLV-1/2 surveillance to gather epidemiological information, and HTLV policies that were implemented independently by states and municipalities. In addition to the COVID-19 pandemic, existing demands from different diseases, the country's demography and its marked sociocultural diversity and the volatility of the technical team working with HTLV-1/2 at the Brazilian Ministry of Health are threats to the implementation of public policies on HTLV-1/2. This SWOT analysis will facilitate strategic planning to allow continuous progress of the Brazilian response to HTLV-1/2 infection.

## Introduction

Human T-lymphotropic virus (HTLV) is a public health issue for most countries. HTLV-1 was identified as the first human oncogenic retrovirus in 1980, and since then, few strategies have been implemented to control it. It is estimated that at least 5–10 million individuals are infected by HTLV-1 globally. However, there are major gaps in the epidemiology of infection, which create difficulties in assessing its public health burden and evaluating policies that favor the prevention and control of HTLV-1/2 (1). The main endemic regions are the southwestern part of Japan, sub-Saharan Africa and South America, the Caribbean area, and the Middle East and Australo-Melanesia (2).

HTLV-1 is the etiologic agent of adult T cell leukemia lymphoma (ATLL), an aggressive neoplasm, and HTLV-1-associated myelopathy (HAM), a chronic severe myelopathy (1, 3). This virus is also associated with infective dermatitis, uveitis, pulmonary lesions, increased mortality (3), and impaired quality of life (4) and contributes to health inequities through its socioeconomic impact (5). Patient care is complex and requires a multidisciplinary approach. The main routes of HTLV-1 transmission are sexual, parenteral and from mother to child (primarily through breastfeeding) (2). There are effective measures for preventing HTLV-1 dissemination, such as screening of blood donors, antenatal screening, counseling, shortening or avoidance of breastfeeding, and promotion of safe sex, but few policies have been implemented in most countries (1). The implementation of public health policies for HTLV-1 in Brazil has been advancing considerably in recent years. However, there are many challenges that need to be overcome to achieve a meaningful response to HTLV-1/2 (6), aiming at the elimination of this virus, as proposed by the Pan-American Organization (PAHO)/World Health Organization (WHO) (7).

## Methods

**Figure 1:**
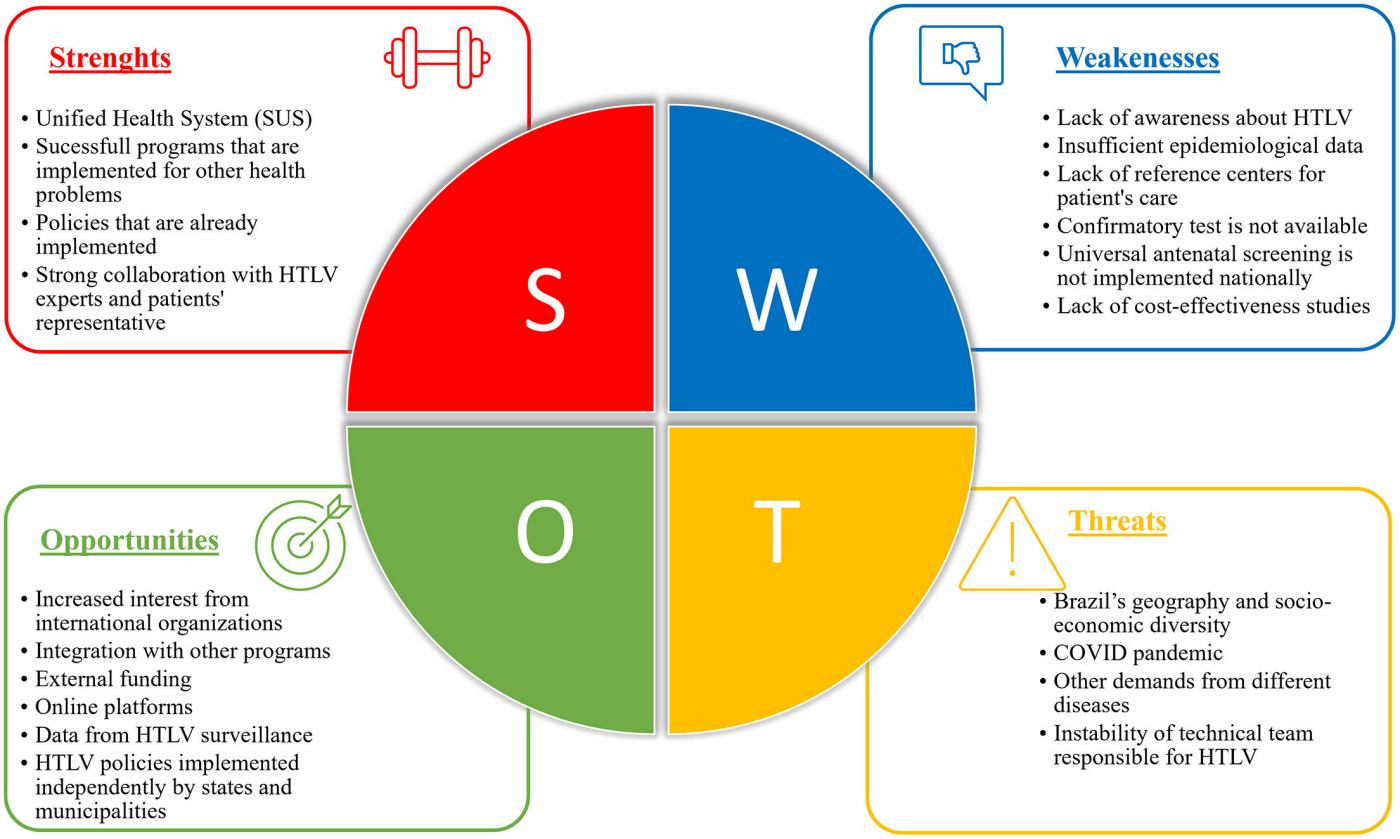
SWOT analysis of the implementation of public policies for HTLV in Brazil.

SWOT analysis is a strategic planning technique that is commonly used in business planning to evaluate an organization's strategic position. The analysis comprises four core elements: strengths, weaknesses, opportunities, and threats. Here, a SWOT analysis is employed to achieve a situational evaluation of the implementation of public health policies for HTLV in Brazil to facilitate future strategic planning (Figure 1).

Based on SWOT Analysis, governments can choose the appropriate strategy. The analysis' process for this study started by identifying the strategic management process and conducting an external and internal analysis. The external analysis identified the critical threats and opportunities for the implementation of HTLV related policies in the competitive Brazilian health scenario. While external analysis focused on the environmental threats and opportunities, internal analysis helped to identify structural strengths and weaknesses associated with HTLV policies and understanding which resources and capabilities are likely to be sources of reasonable advantage.

The analysis was conducted by reviewing Brazilian official documents and papers related to HTLV approaches in Brazil and using researchers, government managers and non-governmental experiences and expertise regarding health policies in Brazil.

## Results and Discussion

### Strengths

Four strengths, i.e., what the Brazilian Ministry of Health (BMoH) excels at, were identified: the Brazilian Unified Health System, successful health programs already implemented in Brazil, specific policies on HTLV-1/2 that are already available in the country and the strong collaboration with HTLV experts and patient representatives.

#### (a) Brazilian Unified Health System

The country has a decentralized, universal public health system, the Unified National Health System (*Sistema Único de Saúde*, SUS). The SUS was proposed in the late 1980s and has been widely acknowledged as an example of successful health system reform in Latin America. It was structured on three principles: universality, comprehensiveness, and equity (8). Brazil is one of the countries that has a varied and well-established care network, ranging from care for people with general illnesses through a network of screening, counseling, care and monitoring of pregnant women to the unique experience of applying sequential and universal immunobiological care, as required by national vaccination programs against polio, measles, and hepatitis B, among others. Reforms in health system governance and major expansion of primary healthcare have contributed to major improvements in health service coverage and access to better health outcomes (9–11). However, disparities in access to effective care persist (12, 13), and the private sector, represented by private health insurance, has been introduced as a supplement to the public health system in many regions.

Primary health care, the service that is responsible for local care in Brazil, is the preferential entry for persons with HTLV-1/2 into SUS, acting in an integrated manner with specialized services, with well-defined reference and counterreferral flows with secondary and tertiary levels of attention. The expansion of primary care in Brazil has been linked to a decline in unnecessary hospitalization (11). Secondary referral or specialized care services are able to provide multidisciplinary care, including psychological support and physiotherapy, and can be used for patients with HTLV-1-associated diseases. Timely treatment can minimize or delay disease progression. Specialized health services are also able to identify patients infected by HTLV-1/2, such as patients with leukemia and lymphomas or patients with other HTLV-1-associated diseases.

#### (b) Successful Health Programs Implemented in SUS

Public health in Brazil is structured within the SUS, where ~75% of the Brazilian population is formally seen during the first stage of health access. In this context, the BMoH has priority actions to address some public health issues, including the Family Health Strategy, which is the gateway to the SUS; the National Immunization Program; mobile emergency care services (SAMU 192); the “Mais Médicos Program,” recently renamed to “Doctors for Brazil,” which aims to meet a demand for the absence or scarcity of health professionals in regions with populations of higher vulnerability; the national system for organ and bone marrow donation and transplantation; the psychosocial care network; and the Popular Pharmacy Program that aims to supply essential medicines. In addition to the drugs offered by the popular pharmacy, there are drugs that are considered strategic and used for the treatment of diseases with an endemic profile that have a socioeconomic impact, such as for STI/AIDS (antiretrovirals, penicillin and condoms), endemic diseases (malaria, leishmaniasis, Chagas disease, leprosy, tuberculosis), thalidomide for systemic lupus erythematosus, multiple myeloma, hematological diseases and blood products, influenza and drugs and supplies for tobacco control (13).

The SUS has also presented some strategies for professional development and health education. Telessaúde (Tele-health Program) is a nationwide initiative that seeks to improve the quality of care and primary care in the SUS, integrating education and service through information technology tools, which offer conditions to promote tele-assistance and tele-education for health professionals. Additionally, the Open University System of the SUS (UNA-SUS) was implemented for professional training and continuing education needs. This system has a collaborative network that includes more than 35 higher education institutions that offer free online courses. UNA-SUS has more than 4.9 million enrollments, and more than 355 courses are offered nationwide (https://www.unasus.gov.br/). From this same perspective, the Virtual Learning Environment of the Unified Health System (AVASUS), which is also a virtual learning space developed for professionals and students in the health area and for civil society, has been implemented, and its main objective is to improve training, management and assistance in the SUS. AVASUS has ~2 million registrations, and a total of 315 active courses. One of them is the Course on Comprehensive Care for People with Sexually Transmitted Infections, which was a partnership with BMoH, PAHO and the Federal University of Rio Grande do Norte. The course is presented with subtitles in Spanish and English and includes a module about HTLV infection (https://avasus.ufrn.br/).

#### (c) Health Policies on HTLV-1/2 Implemented in the Country

**Figure 2:**
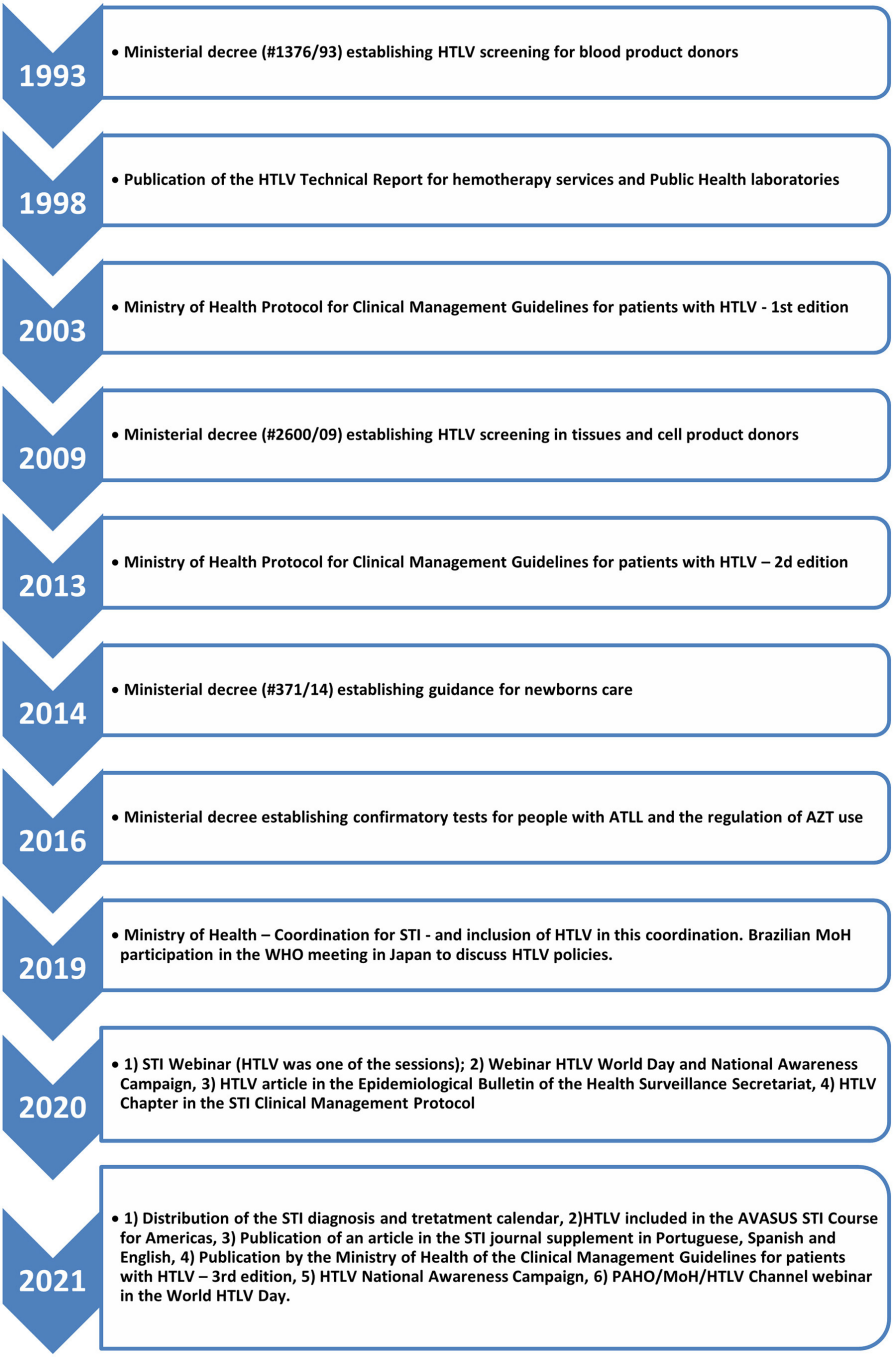
Timeline of the implementation of public policies for HTLV control in Brazil.

Figure 2 summarizes the public policies adopted in the country regarding the control of HTLV infection in Brazil. HTLV-1/2 infection screening has been routinely included in blood banks since 1993 (14) and in organ or tissue recipients and donors since 2009 (15). In 2014, a ministerial decree (#371/14) was published establishing guidance for newborn care, and in 2016, another ministerial decree established confirmatory tests for people with ATLL and the regulation of AZT use (16). The first edition of the Ministry of Health Protocol for Clinical Management Guidelines for patients with HTLV was published in 2003 (17); the second, in 2013 (18); and the third, in 2021 (19). BMoH recommended the use of 0.5 mg cabergoline and infant formula to prevent vertical transmission of HIV and HTLV in 2021 (20).

The integration of HTLV-1/2 into the Department of Chronic Diseases and STI centralized the response to this virus and has been beneficial to the implementation of successful strategies in recent years.

#### (d) Strong Collaboration With HTLV Experts and Patient Representatives

The Department of Chronic Diseases and Sexually Transmitted Infections in the BMoH has a long history of collaboration with HTLV experts and patient representatives. This has been reinforced in recent years and has resulted in meaningful achievements, comprising HTLV awareness campaigns, training of healthcare professionals, production of technical material such as manuscripts on HTLV (21, 22), the HTLV epidemiological bulletin (23) and the HTLV Clinical Guideline (19). HTLV experts and patient representatives also support the BMoH in identifying priorities for action and in closing gaps in the understanding of the burden of HTLV in the country.

### Weaknesses

The weaknesses identified included lack of awareness about HTLV infection, insufficient epidemiological data, lack of specialized reference centers for HTLV care, lack of confirmatory tests, lack of implementation of HTLV-1/2 universal antenatal screening, and lack of cost effectiveness studies.

#### (a) Lack of Awareness About HTLV

One of the major obstacles to implementing policies for HTLV-1/2 is the lack of awareness about these viruses. This contributes to delayed diagnosis of infection, which, in turn, has severe consequences, such as missed opportunities for preventing transmission and delayed treatment, which contributes to poor therapeutic response. In addition, lack of awareness contributes to increased stigma and feelings of abandonment (24–26). Patient advocacy groups may play a major role in this regard but are limited in the country. A recent study showed that HTLVida, a patient advocacy group from Bahia, Brazil, played a central role in the implementation of policies on HTLV in Bahia (27).

HTLV is a silent infection, and many patients are unaware of their serostatus. It is important to increase the visibility of this infection and generate data to inform health professionals and managers about the different aspects of the infection, including its associated diseases, forms of transmission, diagnosis and treatment, with information on how to monitor people living with HTLV in Brazil (19). Increased awareness is important to support proposals for primary prevention and secondary and tertiary HTLV care in the scope of the SUS. Awareness about this infection among the general population is pivotal for the successful inclusion of HTLV in the governmental agenda.

#### (b) Insufficient Epidemiological Data

The national prevalence of HTLV-1/2 is not well-known in Brazil, but regional studies show that it is significant but not homogenous throughout the country. Situational diagnosis of the infection is important to elaborate significant policies. An epidemiological bulletin about HTLV prevalence and distribution in the country was published by the BMoH in collaboration with HTLV experts (23). Most information comes from specific populations, such as blood donors, pregnant women, patients with HTLV-1/2-associated diseases, relatives of infected individuals, and specific groups, such as injectable drug users (IDUs) and sex workers. Although none of these populations represent the general population, the pooled analysis of these data allowed the identification of HTLV as a matter of concern in the country. Therefore, insufficient epidemiological data are still considered a weakness for the implementation of policies to prevent and control HTLV infection in Brazil. Recent and reliable information is needed to adapt and implement targeted public health measures (28). The lack of a register system contributes to the lack of epidemiological data on HTLV-1/2. Although it is possible to obtain information about HTLV-1/2 from some national information systems, such as tests prescribed or complications of outpatients and hospitalized patients, these data are focused on providing information for financial purposes and not for infection surveillance. Notification systems have been useful to the response to HTLV and to better understand disease pathogenesis in Japan and in the UK (29, 30).

#### (c) Lack of Reference Centers for Patient Care

Another challenge is providing reference centers with adequate health care for patients with HTLV in Brazil. Assistance is spread across different clinics, and patients do not have a referral service that can provide adequate care. There is a need to create centers for people suspected of being infected with HTLV-1/2, offering counseling regarding diagnosis, clinical and psychological support, and treatment of associated diseases, including rehabilitation, when necessary.

**Figure 3:**
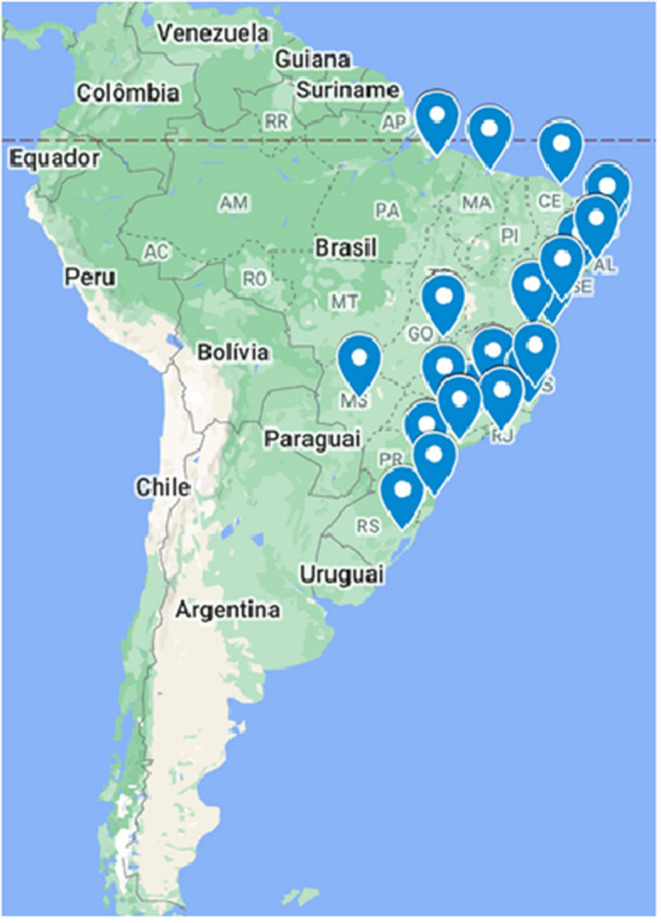
Distribution of centers that offer care for people living with HTLV-1/2 in Brazil.

The BMoH performed a situational diagnosis and mapped all centers that are currently providing HTLV care in the country. There are 33 medical centers providing care for people living with HTLV-1/2 in the country, some of which are linked to public and private universities and offer comprehensive multidisciplinary care, including access to complex laboratory tests that are necessary for the care of people living with HTLV. However, less than half (44%) had laboratory services to support patient care, and only nine had a multidisciplinary team. There is also an unequal distribution of those centers, with the lowest numbers located in the North region (Figure 3). It is important to mention that some centers receive federal funds from SUS for providing clinical, laboratory, treatment, follow-up, and supportive psychological and physiotherapy, when available.

There is a need to improve the support for existing services and create new ones in areas where necessary. A first step should be the establishment of local and regional reference centers for laboratory diagnosis for HTLV-1/2, taking advantage of the national network of Public Health Central Laboratories (LACEN) and the National HIV-1 and hepatitis B (HBV) networks. A hub-spoke system, such as that observed for patient care in services linked to universities that are similar to the Nacional Centre of Human Retrovirology (NCHR) in the UK (http://htlv.eu/) (31), could be a feasible and interesting model to be implemented in all regions of Brazil.

#### (d) Confirmatory Tests Are Not Available for all Seropositive Patients

Confirmatory testing for HTLV-1/2 infection is needed but is not offered for all individuals with a positive result on the screening test (6, 32). PCR and Western blot are included in the SUS to confirm infection but are limited for those with clinical suspicion of ATLL (16). This hampers the identification of the actual burden of infection and prevents adequate counseling, patient management, and control of virus transmission. Confirmatory testing is not compulsory at blood banks. Indeed, the BMoH recently performed a survey and observed that only 40% of the blood centers that participated in the survey offer confirmatory tests for HTLV-1/2. This represents an important weakness of the Brazilian response to this virus, as the same survey identified that only 42% of reactive samples were further confirmed. In contrast, most blood centers (93.3%) reported a link to other institutional services to provide patient counseling, testing and care.

#### (e) Universal Antenatal Screening Is Not Implemented Nationally

Prevention of mother-to-child transmission is considered a priority; however, antenatal national screening has not been implemented nationally in Brazil. The BMoH recommends cabergoline for seropositive mothers and provision of formula for babies who are born to seropositive mothers (20). Optimal clinical management of pregnant women throughout the country was proposed recently (6). Although antenatal screening is already implemented in some Brazilian states, such as Bahia, Mato Grosso do Sul and the Federal District, it needs to be expanded to a national level. Japan alone has a nationwide antenatal screening program, and it is one of the main pillars of the country's response to HTLV (33, 34).

#### (f) Lack of Cost-Effectiveness Studies

Cost-effectiveness analyses are extremely limited in the HTLV-1/2 field and hampered by the lack of key information (35–37) but are important for establishing priorities in public health policies to be implemented in a scenario of limited resources and many demands.

### Opportunities

Different opportunities were identified: increased prioritization by international organizations on HTLV, possibility of including HTLV in other programs, external funding for research, availability of online platforms, opportunity to acquire data from HTLV-1/2 surveillance to gather epidemiological information, and policies on HTLV that have already been implemented by some states and municipalities. Taking advantage of these timely opportunities will foster the implementation of policies on HTLV-1/2 in the country.

#### (a) Increased Priority From International Organizations

International organizations have been showing increased attention to HTLV. In 2019, the WHO held a global consultation on HTLV, which was followed by the publication of a fact sheet (38) and a technical report on HTLV (1). In 2020, the PAHO included HTLV-1 among the pathogens that should be considered for elimination (7), and in 2021, along with the HTLV Channel (a platform to increase awareness about HTLV founded by Brazilian researchers), PAHO co-organized a webinar to discuss public policies on HTLV. The Webinar is fully available at the HTLV channel (https://www.youtube.com/channel/UCI6aLSTtk7chXMeybJ92Fhw).

This increased focus on HTLV-1/2 is catalytical for the implementation of policies by WHO Member States and facilitates the introduction of HTLV-1/2 into the governmental agenda. The HTLV community should take advantage of this opportunity to push for the advancement of public health policies toward this virus.

#### (b) Inclusion of HTLV-1/2 Response in Other Programs

The association of HTLV-1/2 with existing platforms, such as STIs, maternal health, and indigenous health, is an interesting alternative for optimizing resources. In fact, the inclusion of HTLV-1/2 in the Department of Chronic Diseases and STIs was beneficial and should be further put forward as universal knowledge in the country. The inclusion of HTLV-1/2 in awareness campaigns for other STIs is of utmost importance. This represents an opportunity, and policymakers need to take advantage of the network and human resources that are available for those programs to implement public health policies for HTLV. Apparently, there is no other country publishing specific guidelines to prevent sexual transmission of HTLV. In Brazil, HTLV has been included recently in the clinical protocol and therapeutic guidelines for sexually transmitted infections (STI) (6).

#### (c) External Funding for Research

The BMoH promotes scientific and technological development in the country by funding research projects in each of the Brazilian states in partnership with the research program for the SUS. In addition, there are possibilities with the Program for Institutional Development of SUS (PROADI-SUS); this is a program through which non-profit hospital institutions develop projects using their skills to qualify and develop processes to improve the assistance provided by SUS throughout Brazil. Presently, six hospitals of national excellence participate in this program. Additionally, there are national calls for partnerships with other federal ministerial areas and international agencies, including the Ministry of Science and Technology and PAHO. Recently, HTLV was included in two research opportunities that are currently underway. In 2019, the PROADI-SUS approved a project to assess the national prevalence of HTLV and other STIs and patterns of sexual behavior among pregnant women.

In 2021, the BMoH, in partnership with PAHO, co-founded a project conducted by public universities in the north, northeast and midwest regions on the implementation of HTLV laboratory diagnosis in Brazil. This project aims to expand the network for diagnosing HTLV-1/2 infection in six Brazilian capitals and to characterize the types and subtypes of HTLV-1/2 that are circulating in the Brazilian population. The project was initially funded by the National Council for Scientific and Technological Development in the Ministry of Science and Technology and the Brazilian Ministry of Health, initiated in 2019 in a partnership with the State Health Department of Para, Special Indigenous Sanitary District (DESAI) and Special Secretariat for Indigenous Health (SESAI) and 13 other research institutions. A large and comprehensive seroepidemiological study will provide specific and updated information in 11 states, from at least 6,000 persons from 50 population groups residing in urban, rural and indigenous areas. It is expected that at the end, this study will provide a more reliable figure of the prevalence of HTLV-1/2. Vulnerable populations, including quilombola (originally formed by slave escapees), female sex workers (FSW), men who have sex with men (MSM), and indigenous communities, will be investigated, and full support will be provided, including training and information teaching programs for health professionals from the DSEI/SESAI and the preparation of an informational leaflet in indigenous languages. It is expected that such an opportunity will also help DESAI/SESAI to understand the current epidemiological situation of indigenous people.

However, this should be expanded, and collaboration between these institutions and patient representatives and specialists should be supported. Some key areas for research were identified as (1) the development of a rapid, cost-effective point-of-care screening test, (2) the proposal of multicentric studies with validated methodologies for standardizing the diagnosis of HTLV-1/2 in the national territory; (3) the evaluation of HTLV mother-to-child transmission and policies to prevent vertical transmission; (4) cost-effectiveness studies; and (5) the development of new therapies and care for HTLV-1-associated diseases.

#### (d) Online Platforms for HTLV Awareness

**Figure 4:**
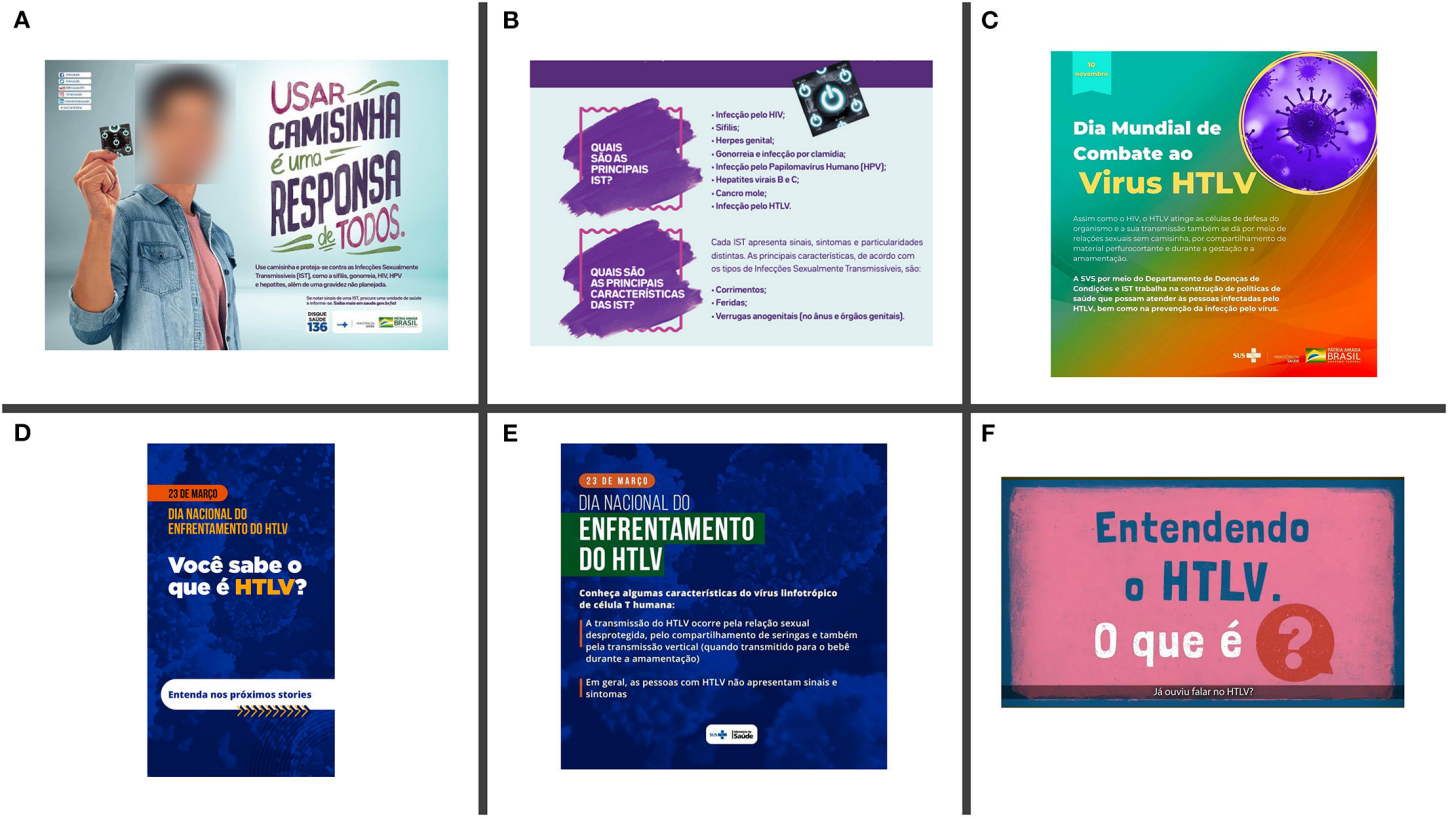
Awareness campaign about HTLV-1/2 promoted by the Brazilian Ministry of Health. **(A,B)** 2019 awareness campaign; **(C)** HTLV National day awareness campaign 2020; **(D,E)** HTLV National day awareness campaign 2021; **(F)** HTLV World Day awareness campaign 2021.

Social media has played an important role in sharing health-related information. In recent years, the BMoH has been increasingly using their social media platforms to advertise about HTLV (Figure 4). There are also an increasing number of online platforms that were created with the aim of raising awareness about this virus, such as the HTLV Channel and HTLVida. HTLVida social media was created by the patient advocacy group from Bahia, and the HTLV Channel was founded by researchers and has been collaborating with the BMoH, for example, for the organization of two webinars in 2020 and 2021 on the occasion of the HTLV World Day [both webinars are fully available at the HTLV Channel (https://www.youtube.com/channel/UCI6aLSTtk7chXMeybJ92Fhw)].

#### (e) Data Acquisition From HTLV-1/2 Routine Surveillance

HTLV-1/2 screening of blood and organ donors has been performed in the country for decades. However, these data have not been routinely used to inform policymakers about HTLV-1/2 prevalence and distribution. Although the burden of infection in this specific population may underestimate the real burden of HTLV-1/2 infection, it can be a starting point for those areas of the country where there are no data available about HTLV-1/2 distribution as has been done recently in China (39).

#### (f) HTLV Policies Implemented Independently by Brazilian States and Municipalities

Different states and municipalities have implemented policies for HTLV-1/2 independent of national financial support. Bahia, considered the epicenter of HTLV-1 infection in Brazil, has approved a program for the integral care of patients with HTLV infection (40). This should be considered a model for other states. It will be helpful to perform a situational assessment of the available local policies on HTLV-1/2 in each Brazilian state. This would facilitate the expansion of policies to a national level and support those local governments that are aiming at starting implementation of such policies in their area.

### Threats

Brazil's demography and sociocultural diversity, demands of different health problems, including the COVID-19 pandemic, and the volatility of the technical team working on HTLV-1/2 at the BMoH are common causes hampering the implementation of policies toward HTLV-1/2 and therefore are considered threats.

#### (a) The Country's Demography and Sociocultural Diversity

Brazil is a large country, with an area of 8.5 million km^2^, with more than 214 million individuals distributed in five different geographical regions. According to the International Monetary Fund the country is considered of medium income. The illiteracy rate of the population aged 15 and over in Brazil is 7.0% (2017). This rate varies across the country, being higher in the North and Northeast regions when compared to the South and Southeast. The country is marked by great sociocultural diversity and inequal income distribution. Its municipalities are highly diverse in terms of population size, structure of local and regional health systems, and the private sector's greater or lesser presence in some regions (13). Regarding the access to internet, Brazil has the fifth largest online population in the world, around seven out of 10 Brazilians are online, and 90% have daily access to the web. This poses additional difficulties when implementing national policies on HTLV. Although national policies are needed, the particularities should be considered when designing health policies for preventing and controlling this virus. This is also of utmost importance when designing policies targeting specific vulnerable populations, such as indigenous communities, sex workers, and people who inject illicit drugs. In addition, this diversity particularly influences the access and effectiveness of care for those living with HTLV-1/2 in the country. Therefore, an effective network with care centers located regionally is needed.

#### (b) Demands of Other Health Problems

As a tropical middle-income country with a population that is aging, there are many demands from other health problems. These include non-communicable diseases (NCDs), neglected tropical infections, and recurrent epidemics. In 2016, NCDs were the leading cause of death, followed by interpersonal violence, but infectious diseases were still common causes (41). In this regard, increasing awareness about HTLV is beneficial for its inclusion in the governmental agenda.

#### (c) The COVID-19 Pandemic

The pandemic caused by SARS-CoV-2 disrupted the health care system in every country worldwide (42). In Brazil, this was no different (43). Preparedness was not enough to face such an explosive epidemic in the country (44). Preventive care declined significantly during the pandemic by approximately 1.4-fold (45), vaccination coverage decreased 20% (46), and research was deeply affected. This threatens the advancement of research on other health issues and care for patients, including those with HTLV-1/2. In Brazil, telemedicine helped to ensure continuous care of patients with HTLV during the COVID-19 pandemic (47) and can be applied to facilitate the implementation of regional specialized reference centers and linked local services.

#### (d) Volatility of the Technical Team Responsible for the HTLV Response

The implementation of policies for preventing and controlling HTLV-1/2 infection are usually long-term actions that require long-term planning. Continuity needs to be guaranteed, despite changes in government parties. There is a need to assure that these policies, which will benefit the population in the short- and long-term period, should be dealt with as a country rather than as a government policy. The volatility of the technical team at the BMoH responsible for the HTLV response is detrimental to the continuous progress of policies on HTLV-1/2 in the country. It is also important to highlight that the volatility of state and municipal health teams in the public system may also impact the implementation of policies. The high turnover of health teams in public clinics, can impair patient's care, as it may be difficult to do a long-term follow-up of HTLV patients, individuals with life-long infection, and who usually present chronic diseases, impacting the quality of the service offered.

## Conclusion

HTLV-1/2 antenatal screening should be considered a priority. The expansion of confirmatory tests to all individuals with a positive screening test is needed. Mapping local policies to HTLV-1/2 in different Brazilian states will be beneficial for evaluating policies that can be easily expanded to a national level. The integration of HTLV-1/2 into existing programs and available networks may be achieved in a short-term period and at relatively low cost. A network comprising regional reference centers and local linked services needs to be organized. Training of healthcare professionals is essential to the success of every proposed policy.

The government needs to continuously work in partnership with the scientific community and non-governmental organizations to increase the visibility of the HTLV infection. It should be interesting to support, whenever possible, initiatives proposed by partners, such as the propagation of National HTLV Day and World HTLV Day, and to expand the information network on HTLV-1/2. The conjunction of efforts between the government, health professionals and the scientific community, in addition to the active participation of organized civil society, provides conditions for adequately addressing HTLV-1/2 infection and its complications in the country.

In conclusion, this analysis may help policymakers to develop a strategic planning with short and long-term goals to improve Brazil's response to HTLV-1/2 infection to facilitate the implementation of recommended policies targeting HTLV that were recently published (6). In addition, this SWOT analysis may be used as a model to be implemented in other scenarios worldwide.

## Data Availability Statement

The original contributions presented in the study are included in the article/Supplementary Material, further inquiries can be directed to the corresponding author.

## Author Contributions

AM, CR, and RI conceptualized the manuscript. AM and CR drafted the initial manuscript. AM, CR, TA, GP, AV, and RI wrote and critically revised the final manuscript. All authors approved the final version of the manuscript.

## Funding

The authors acknowledge the Conselho Nacional de Desenvolvimento Cientifico e Tecnologico, CNPq (Research grants RI #312979/2018-5 and ACRV #301869/2017-0), the Brazilian Ministry of Health, the Pan American Health Organization and the Universidade Federal do Para (UFPA), PROPESP/2022, for their generous financial support.

## Conflict of Interest

The authors declare that the research was conducted in the absence of any commercial or financial relationships that could be construed as a potential conflict of interest.

## Publisher's Note

All claims expressed in this article are solely those of the authors and do not necessarily represent those of their affiliated organizations, or those of the publisher, the editors and the reviewers. Any product that may be evaluated in this article, or claim that may be made by its manufacturer, is not guaranteed or endorsed by the publisher.
