# Supplementary material for: Strengths, Weaknesses, Opportunities and Threats (SWOT) Analysis of the Implementation of Public Health Policies on HTLV-1 in Brazil
Source: Front Med (Lausanne). 2022 Apr 7;9:859115. doi: 10.3389/fmed.2022.859115 (PMC9021745; doi:10.3389/fmed.2022.859115)
Supplement: Supplementary file 1 [file Table_1.DOCX]

Supplementary material

**Table 1. Clinical Care Centers for people with HTLV in Brazil**

| **Service Name** | **City** |  | **State** |  |
| --- | --- | --- | --- | --- |
| Unidade de Doenças Infecciosas e Parasitárias | Maceió |  | Alagoas |  |
| Centro de Neurologia, Escola Bahiana de Medicina e Saúde Pública | Salvador |  | Bahia |  |
| SMS de Maranguape | Manguarape |  | Ceará |  |
| CRIAS | Cachoeira do Itapimirim |  | Espírito Santo |  |
| NAPS - Núcleo de Atenção e Promoção A Saúde | Linhares |  | Espírito Santo |  |
| SAE/CTA | Caldas Novas |  | Goiás |  |
| Hospital Presidente Vargas | São Luiz |  | Maranhão |  |
| SAE/CTA | Cuiabá |  | Mato Grosso |  |
| CTA-SAE - Ambulatorio | Tangará da Serra |  | Mato Grosso |  |
| Hospital Universitário Maria Aparecida Pedrossian | Campo Grande |  | Mato Grosso do Sul |  |
| Policlínica Municipal de Divinópolis | Divinópolis |  | Minas Gerais |  |
| Centro de Especialidades Multiprofissional - CEM | Barbacena |  | Minas Gerais |  |
| Hospital Eduardo De Menezes -FHEMIG | Belo Horizonte |  | Minas Gerais |  |
| SAE/CTA/UDM - Ambulatorio Herbert De Souza | Uberlândia |  | Minas Gerais |  |
| São Sebastião do Paraíso - Ambulatório de Infectologia | São Sebastião do Paraíso |  | Minas Gerais |  |
| Unidade Complexa William Rocha | Belo Horizonte |  | Minas Gerais |  |
| Secretaria Municipal De Saúde | Belo Horizonte |  | Minas Gerais |  |
| Secretaria Municipal De Saude | São João Del Rei |  | Minas Gerais |  |
| Centro de Testagem e Aconselhamento / Serviço de Atenção Especializada | Três Pontas |  | Minas Gerais |  |
| Secretaria de Saúde de Varginha - Policlínica Central | Varginha |  | Minas Gerais |  |
| Secretaria de Saúde de Passos | Passos |  | Minas Gerais |  |
| Laboratório de Virologia - Instituto de Ciências Biológicas | Belém |  | Pará |  |
| Universidade Federal do Pará / Núcleo de Medicina Tropical | Belém |  | Pará |  |
| Hospital Gafree e Guinle | Rio de Janeiro |  | Rio de Janeiro |  |
| Hospital Universsitário Pedro Ernesto | Rio de Janeiro |  | Rio de Janeiro |  |
| INI-FIOCRUZ-RJ | Rio de Janeiro |  | Rio de Janeiro |  |
| Complexo Hospitalar Universitário Prof. Edgard Santos | Canela |  | Rio Grande do Sul |  |
| Hospital de Clínicas De Porto Alegre | Porto Alegre |  | Rio Grande do Sul |  |
| HCSC - Jataí | Jataí |  | Santa Catarina |  |
| Hematologia - HCFMUSP | São Paulo |  | São Paulo |  |
| Vigilancia Epidemiologica | São José dos Campos |  | São Paulo |  |
| CEDIC CTA | Valinhos |  | São Paulo |  |
| Ambulatorio de Infectologia de Tatui | Tatuí |  | São Paulo |  |
| Sae Carlos Borges Ancora da Luz | Tremembé |  | São Paulo |  |
| Policlínica Centro | São Bernardo do Campo |  | São Paulo |  |
| Ambulatório De Especialidades Médicas Jerônimo Ribeiro | Francisco Morato |  | São Paulo |  |
| CEMEG Centro Guarulhos | Guarulhos |  | São Paulo |  |
| Instituto de Infectologia Emilio Ribas | São Paulo |  | São Paulo |  |
| Ambulatório da Criança | Guarulhos |  | São Paulo |  |
| Serviço de Atendimento Especializado IST's, HIV/ Aids E Hepatites Virais | Sumaré |  | São Paulo |  |
| CTA SAE de Infectologia de Osasco | Osasco |  | São Paulo |  |
